# Comparative analysis of the efficacy and complications of mid-urethral slings when inserted either in isolation or in conjunction with pelvic organ prolapse surgery

**DOI:** 10.1007/s00404-024-07691-z

**Published:** 2024-09-08

**Authors:** Esther Ruiz Pérez, Sonia De Miguel Manso, Elena García García, Julio Alberto Gobernado Tejedor, Álvaro Sanz Díaz-Heredero, Lidia Casamayor Del Nogal, Sandra Canales Martínez, Jimena Bayón Pascual

**Affiliations:** 1 Department of Obstetrics and Gynecology, Hospital Clínico Universitario of Valladolid, Regional Health Management of Castilla y León (SACYL), Avenida Ramón y Cajal 3, 47005 Valladolid, Spain; 2 Department of Pediatrics and Immunology, Obstetrics and Gynecology, Nutrition and Bromatology, Psychiatry, and History of Science, Faculty of Medicine, University of Valladolid, Valladolid, Spain

**Keywords:** Stress urinary incontinence, Pelvic organ prolapse, Total urinary continence, Transobturator mid-urethral sling

## Abstract

**Introduction:**

Stress urinary incontinence (SUI) is a highly prevalent condition that affects between 20 and 50% of the female population. Pelvic organ prolapse (POP) can coexist with SUI and both can be addressed through a vaginal approach. However, it is unclear whether simultaneous surgery for these two conditions can influence the outcome of incontinence treatment.

**Objective:**

To evaluate the objective and subjective effectiveness of the transobturator suburethral (TO) band by comparing two groups: group A, of patients undergoing surgery for stress urinary incontinence (SUI) by insertion of TO mesh, and group B, formed for patients requiring simultaneous correction of pelvic organ prolapse (POP) in addition to TO mesh insertion.

**Materials and methods:**

This is an observational, descriptive and retrospective study in which 91 patients participated: 33 (group A) underwent surgery for SUI and 58 (group B) underwent corrective surgery for pelvic organ prolapse (POP) and TO band simultaneously.

Variables included: total urinary continence, objective urinary continence, subjective urinary continence (satisfaction levels and two validated questionnaires (PGI-1 and ICIQ-SF)) and complications.

**Results:**

Regarding total continence, from the seventh to the ninth year, statistically significant differences were observed, with total continence being higher in group A. Objective continence decreased in both groups during the follow-up period.

No significant differences were found between the two groups regarding subjective urinary continence (ICIQ-SF) and the degree of improvement after surgical treatment (PGI-1). The level of satisfaction after surgery was high in both groups. Regarding complications, there were no statistically significant differences.

**Conclusions:**

Isolated surgery for SUI could be considered more effective in achieving total and objective continence. However, the insertion of the TO band in both cases improves subjective urinary continence and quality of life with great safety and without differences regarding complications.

## What does this study add to the clinical work

**Table:** 

We would like to enrich the number of available studies and expand the literature on isolated surgery for stress urinary incontinence or concomitant surgery for pelvic organ prolapse and SUI, making it accessible to all interested researchers.	

## Introduction

Stress urinary incontinence (SUI), defined as the involuntary loss of urine associated with effort, physical activity, sneezing or coughing, is a very common entity that affects between 20 and 50% of the female population, being more frequent in people over 65 years of age [1].

On the other hand, pelvic organ prolapse (POP) is defined as the descent of one or more organs from their usual anatomic position as a result of inadequate functioning of the support structures. It is estimated that in 40–60% of patients [2], SUI may coexist with POP. Both pathologies share some etiological factors, mainly vaginal delivery, in addition to other recognized risk factors such as obesity, constipation or previous pelvic surgery.

The treatment of SUI must be done in a stepwise manner. Initial management is based on conservative measures that include lifestyle changes such as weight loss, quitting smoking, performing pelvic floor exercises, and bladder reeducation. Within conservative management, the application of vaginal estrogens can contribute to improving the genitourinary syndrome, thus improving incontinence3. Pharmacologic treatment, which includes drugs such as ephedrine, imipramine or alpha-adrenergic agonists, has shown little effectiveness. Duloxetine, however, has been shown to be an effective and safe treatment in the treatment of SUI [3, 4].

When these conservative measures fail to alleviate SUI, or if the patient rejects this initial management, surgical treatment is indicated. The current gold standard in SUI surgery is the insertion of a tension-free synthetic mid-urethral sling. Both POP and SUI can be successfully corrected through a vaginal approach. However, it is controversial whether concomitant surgery for both pathologies can affect the outcome of SUI treatment [2, 5].

The published studies in which SUI and POP surgery are performed in a single intervention indicate similar effectiveness to those studies that propose surgery in two stages. On the other hand, some publications show that POP correction can successfully treat SUI in 30–50% of patients, so we would be overtreating SUI without being necessary and surgery could be performed in two stages if SUI persists later [2, 4].

## Objectives

### Main

To assess the objective and subjective efficacy of the transobturator mid-urethral band (TO) comparing two groups: group A made up of patients who undergo surgery only for SUI with TO mid-urethral band and group B made up of patients who require simultaneous correction of POP symptomatic and SUI, so they associate TO [6].

### Secondary

Check if there are differences in the complications of the procedure between both groups: the group A of patients with isolated TO insertion and the group B of patients who are operated concomitantly with TO and POP surgery.

## Material and methods

A retrospective descriptive study was carried out, in a single center, in the Gynecology and Obstetrics Service of the University Clinical Hospital of Valladolid (Spain), in women undergoing SUI using transobturator band (TO) with both inside-out insertion (in- out) and from outside to inside (out-in) during a period of 10 years (January 2012–December 2022).

The inclusion criteria were that the patient had SUI, whether or not associated with mixed urinary incontinence (MUI), with predominance of SUI symptoms, a positive cough stress test with urethral hypermobility, with or without simultaneous corrective surgery for POP. In the group A, patients without associated POP surgery were included, except if a perineal tear correction (perineorrhaphy) was performed. Patients in group A could have POP or not. If so, the POP was mild and did not require surgical correction, therefore, in these patients only the transobturator band was indicated to treat SUI.

The following exclusion criteria were applied: diagnosis of neurogenic bladder, SUI due to intrinsic urethral sphincter deficit, and postvoid residual > 100 ml. Age and body mass index (BMI) did not limit inclusion in this study.

The patients were initially evaluated through clinical history, physical examination and ultrasound, to obtain anthropometric data, obstetric history and type of urinary incontinence (SUI, MUI). Data were obtained from validated questionnaires such as ICIQ-SF and PGI-1 to assess the degree of incontinence and the impact on quality of life, in addition to the improvement after treatment in our patients, respectively.

During the first 2 years, follow-up was carried out in the Pelvic Floor Unit consultation through physical examination, cough test, measurement of residual urine and assessment of subjective healing through the degree of personal satisfaction and the ICIQ-SF questionnaires and PGI-1. Subsequently, an annual follow-up was established by telephone interview, taking an exhaustive anamnesis on the symptoms related to SUI, urgency and urgency urinary incontinence, assessing the same variables and questionnaires.

The main variables evaluated after surgery were:

- Total urinary continence (which includes the urgency and effort components).
- The objective cure rate (through a negative cough test in consultation or anamnesis conducted in the telephone interview).
- The subjective cure rate was assessed in three ways: through the degree of satisfaction (analog scale from 0 to 10, with scores between 7 and 10 considered very satisfied) and two validated questionnaires, the impression of improvement after treatment (PGI-1 , scale 1–7) and the short version of the urinary incontinence questionnaire of the International Continence Society (ICS), ICIQ-SF (sum of three items, whose result varies between 1 and 21).
- Complications were divided into: immediate (< 7 days), intermediate (≥ 7–30 days) and late (≥ 1 month). The SIU recurrence was defined as the beginning of SUI after a continence period of ≥ 3 months, with a frequency of ≥ 1 time per week, and it was evaluated in both groups, A and B

As for intraoperative checks, cystoscopy is not performed since the sling is transobturator. What is done is catheterizing the patient at the beginning of the surgery, and later, just before placing the sling, it is verified that she is properly catheterized and that the bladder is empty.

In our study, after prolapse surgery was performed in group B, intraoperative controls were not performed to check whether SUI persisted. Because the patient is anesthetized, either generally or regionally, and we do not find the result of performing an artificial Valsalva reliable or reproducible under these conditions. Previously in consultation, before indicating surgery, the cough test is performed with POP and reducing it with a valve, to check if there is SUI with and without POP. Concomitant surgery for POP and SUI was indicated in patients in whom, after reducing POP in consultation, SUI appeared or persisted.

All study variables were included in an Access database, composed of all patients undergoing TO banding since 2012, where their characteristics and follow-up information are noted.

Statistical analysis: continuous quantitative variables with a normal distribution are described by mean and standard deviation, and those with a non-normal distribution by median and interquartile range (P25–P75). Qualitative variables are described by n and percentage. The statistical software used was SSPS v. 23.

### Ethical approval

Authorization of this study by the Ethics and Research Committee of the Health Area to which the center belongs (PI 22–2901). All patients included in the study, upon signing the informed consent for the surgical intervention, were informed of the possibility of transferring their data for research purposes. In the consultation review of the second year, they are informed that they will be called annually for follow-up.

## Results

During this period of time, 91 patients were included in the study, 33 patients in whom only SUI correction was performed with TO band (group A) and 58 in whom POP correction surgery and TO band placement were performed. at the same time (group B). First, the prolapse surgery is performed, and then the mid-urethral sling is placed. The only precaution is to make the incision for the prolapse a bit further away from the urethral meatus, so that there is space afterward to make the incision and place the TOT.

In group A, the bands used were: 30 out-in Monarc^™^ (AMS, Minnetonka, US) and 3 in–out IngyneS (Dipromed^®^, Torino, Italy).

In group B, all the bands that were placed were in–out: 10 IngyneS (Dipromed^®^, Torino, Italy), 10 Gynecare^®^ (Ethicon, US) and 38 I-Stop^®^ (Braun).

Regarding the clinical characteristics of the patients (Table 1), the median age was 66 in group A vs 71 in group B, this being the only characteristic in which a statistically significant difference was obtained (*p* = 0.041). The mean BMI was 27.7 for A and 26.4 for B. The majority of patients in both groups had one or more births (97% A vs 93% B). The median baseline score on the ICIQ-SF questionnaire was 17 in A and 16 in B (without statistically significant differences).

**Table 1:** Clinical characteristics of the patients

Variable	A	B	P
Middle age (range)	66 (38)	71 (44)	Mann–Whitney *U* test 0.041
Mean BMI ± SD	27.7 ± 4.4	26.4 ± 3.4	
Parity			
Nulliparous	3%	6.9%	
≥ 1 vaginal birth	97%	93.1%	
UI type			
SUI	48.5%	50%	
MUI	51.5%	50%	

Data marked in red are statistically significant results

*SD* Standard deviation, *UI* Urinary incontinence

**Fig. 1:**
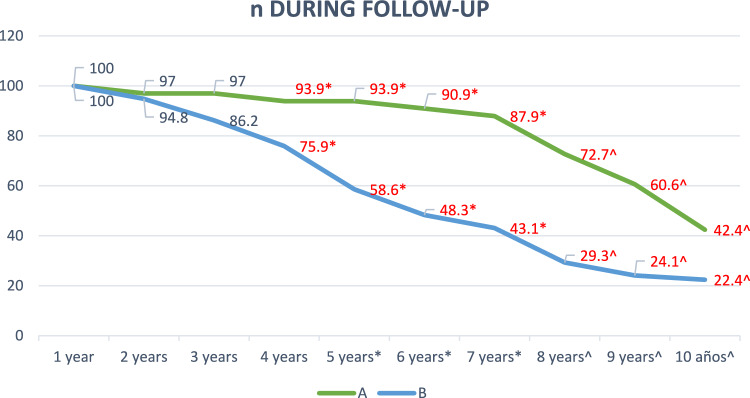
Percentage of patients who undergo follow-up during the first 10 years after the intervention. *p < 0.005 Fisher’s exact p, ^*p* < 0.005 Pearson’s Chi-square. The years of follow-up with statistically significant results are indicated in red

The number of patients who completed follow-up each year is shown in Fig. 1, exceeding 50% during the first 9 years in group A and during the first 5 years in group B.

In group B, the surgeries associated with the insertion of the TO band were: 32 vaginal hysterectomies (only in these patients the uterus was removed), 52 anterior colporrhaphies, 27 posterior colpoperineorrhaphies, 2 enteroceles, 1 colpocleisis, and 45 perineorrhaphies.

**Fig. 2:**
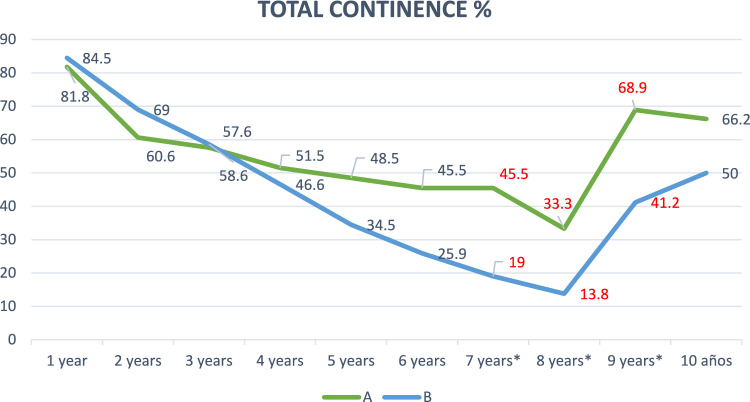
Total continence in groups A and B over the 10 years after surgery. Statistically significant percentages highlighted in red (Pearson’s Chi-square test)

Regarding total continence, which includes urine loss due to both urgency and effort, a striking decrease was observed throughout the 10 years of follow-up, with percentages greater than 80% in the first year in both groups, and a decrease to 33% in group A and 14% in group B at the eighth year. From the seventh to the ninth year, statistically significant differences were obtained between both groups, with total continence being higher in group A (Fig. 2).

**Fig. 3:**
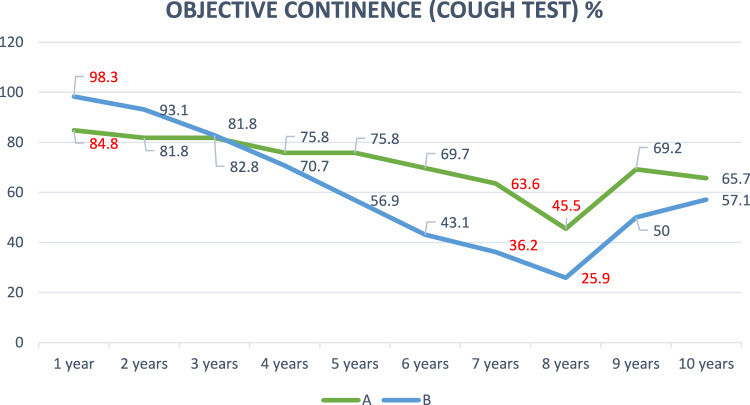
Objective continence in %, assessed by cough test, over the 10 years after the intervention. Statistically significant percentages highlighted in red (Pearson’s Chi-Square test)

Objective continence, which was assessed using the cough test, also decreased in the groups throughout follow-up. In group A, continence greater than 60% was maintained practically during the 10 years, while in group B, objective continence was only maintained above 60% during the first 5 years. The differences between the two groups were statistically significant in 3 years, in the first year in favor of group B and in the seventh and eighth years in favor of A (Fig. 3).

**Fig. 4:**
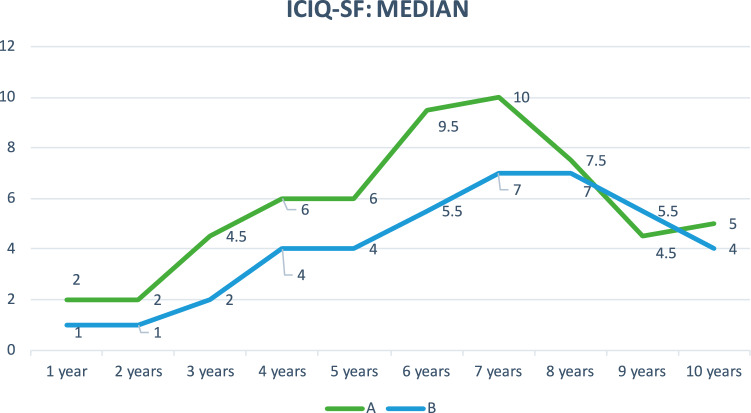
Assessment of quality of life with the ICIQ-SF questionnaire (median) in both groups, A and B, during follow-up

**Fig. 5:**
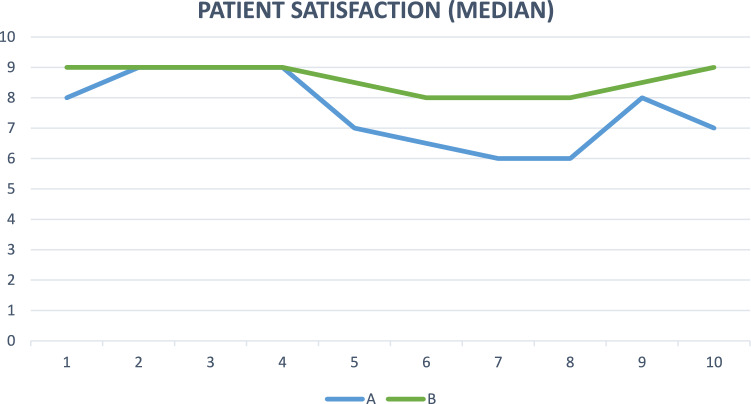
Patient satisfaction (median) in group A and group B during follow-up

**Fig. 6:**
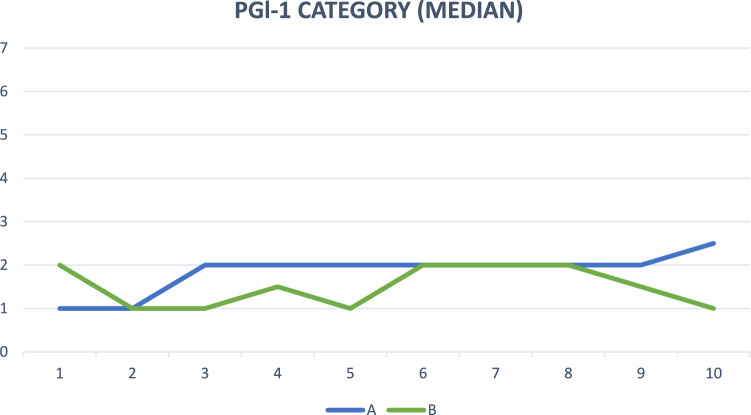
Improvement after treatment of patients (PGI-1, median) in group A and B during follow-up

Regarding subjective healing, based on the degree of patient satisfaction (0–10 scale), the improvement in quality of life (ICIQ-SF) and the degree of improvement after surgical treatment (PGI-1, scale 1–7), no significant differences were found between groups (Mann–Whitney *U* test). Figures 4, 5, and 6.

The score on the ICIQ-SF questionnaire throughout the 10 years of follow-up (Fig. 4) was similar in the two groups, without statistically significant differences being obtained, with a maximum difference of four points between the medians of the two groups at 6 years.

The degree of satisfaction (Fig. 5) was high in both groups A and B throughout the follow-up, with a median score always above 6, with group B being greater than 8 during the 10 years.

Regarding complications, there were no statistically significant differences after treatment between both groups. Table 2 shows the complications that were assessed; urinary retention (in the first week and in the first month), pain from the first month after surgery and recurrence of SUI. Recurrence was defined as the beginning of SUI after a continence period of ≥ 3 months, with a frequency of ≥ 1 time per week, being 21% in A vs 10% in B (*p* > 0.005).

**Table 2:** Complications after surgical intervention in A and B

	A	B
Urinary retention < 7 days	12.1%	10.3%
Urinary retention 7–30 days	3%	3.4%
Pain > 30 days	15.2%	8.6%
SUI recurrence	21.2%	10.3%

## Discussion

Total and objective urinary continence decrease in both groups over 10 years (total continence does more), and these rates are significantly higher in some years in patients in whom only the insertion of the TO is performed (better total continence compared to group B from the seventh to ninth year and better objective continence of group A in the first, seventh and eighth years). This difference could be due to the younger age of the patients in group A.

No differences were found in the degree of improvement after treatment (PGI-1) or in patient satisfaction. In both groups these premises were very good.

In both groups, the quality of life assessed by the ICIQ-SF questionnaire worsens during follow-up, but does not reach the score before surgery (median maximum scores at the seventh year of 10 in group A and 7 in group B, and prior to surgery there were 17 in group A and 16 in group B).

In a prospective study [5] in which the subjective and objective results at 5 years of midurethral TO band placement as a single surgery were compared with a group of patients in whom POP correction surgery was also associated, the subjective and objective cure rates for both groups were 70.5% vs 94.1% (*p* < 0.01) and 80.3% vs 85.7% (*p* = 0.58), respectively. These results, in which objective cure is significantly higher in the group that combines prolapse surgery, do not agree with those obtained in our study. In a retrospective work by Joanna et al. [1] a slightly higher and statistically significant subjective cure rate was obtained in the TOT group with POP surgery (89.6% vs 82.6%; *p* = 0.035). Fatih et al. [2], in their prospective study, observed that there was no statistically significant difference in the objective continence rate if SUI surgery with TO band was associated with POP surgery. In a publication in which the success and complication rates were prospectively evaluated in 72 patients with both simultaneous surgeries, they observed an objective cure rate of 80.6% after one year, which was considerably lower than that obtained in our study (98%). Patients with fewer pregnancies and vaginal deliveries had significantly higher success rates.

There were no significant differences regarding complications after treatment between both groups, A and B, neither overall nor by period, nor when studying specific complications (pain, urinary retention and SUI).

Regarding postsurgical complications, in the work of Joanna et al.1, postoperative urinary retention was more frequent, with a statistically significant difference, in the joint surgery group compared to the group with only TO (18.6% vs 3.2%; *p* < 0.001). Age and obesity were identified as independent factors of TO failure, while interestingly, prolonged postoperative urine retention was a positive predictive factor of TO success.

Fatih et al. [2] observed that patients in the group with both surgeries had greater postsurgical pelvic pain (*p* = 0.012) and a significantly greater postvoid residual volume (*p* = 0.020). In a retrospective study with a 2 year follow-up, in TO procedures with concomitant prolapse repair, a higher incidence of voiding dysfunction was observed in the immediate postoperative period, although it did not persist at the 6 week follow-up visit. There were no differences in reintervention rates [7].

## Conclusions

1- It could be considered that isolated SUI surgery, without associating POP correction, would be more effective in terms of total and objective continence, but the insertion of the TO band in both cases improves subjective healing and quality of life. with great safety and without differences in terms of complications, neither immediate nor late.

2- Studies with a larger number of patients, homogeneous groups and long-term follow-up would be necessary to contrast our results, and to be able to accurately describe the convenience of associating POP surgery or not with the surgical correction of SUI with TO.

## References

[1] Banas J, Jankiewicz K, Rechberger T, Kołodyńska A, Bogusiewicz M (2023) Outcome of transobturator sling for treatment of female stress urinary incontinence applied as a single procedure or concomitantly with pelvic organ prolapse surgery. Annals Agric Environ Med: AAEM 30(1):190–42 10.26444/aaem/162800 36999874

[2] Celik F, Pektas MK, Kose M, Arioz DT, Yesildager E, Yilmazer M (2018) Two-year follow-up results of transobturator tape procedure with and without concomitant vaginal surgery. Urologia Int 100(4):402–8 10.1159/000488465 29627828

[3] SEGO (2018) Diagnóstico de la incontinencia Urinaria de Esfuerzo. In: SEGO, ed. Guías de asistencia práctica en Suelo pélvico

[4] SEGO (2017) Tratamiento de la incontinencia Urinaria de Esfuerzo. In: SEGO, ed. Guías de asistencia práctica en Suelo pélvico

[5] Department of Obstetrics and Gynaecology TCU of HK Prince of Wales Hospital, Shatin Hong Kong, Law TS, Cheung RY, Chung TK, Chan SS (2015) Efficacy and outcomes of transobturator tension-free vaginal tape with or without concomitant pelvic floor repair surgery for urinary stress incontinence: five-year follow-up. Hong Kong Med J Xianggang yi xue za zhi 21(4):333–8 26183453 10.12809/hkmj144397

[6] Ayhan A, Dogan NU, Guven S, Guler OT, Boynukalin FK, Salman MC (2009) Clinical outcome of transobturator tape concomitant with vaginal hysterectomy plus anterior posterior colporrhaphy. Arch Gynecol Obstet 280(3):375–80 19148661 10.1007/s00404-008-0920-0

[7] Houwing MM, Schulz JA, Flood CG, Baydock S, Rosychuk RJ (2013) A retrospective review of tension-free vaginal tape/transobturator tape procedures done concomitantly with prolapse repair. J Obstet Gynaecol Can 35(4):340–7 23660042 10.1016/S1701-2163(15)30962-2

